# Directional Solidification Microstructure of a Ni-Based Superalloy: Influence of a Weak Transverse Magnetic Field

**DOI:** 10.3390/ma8063428

**Published:** 2015-06-10

**Authors:** Xu Li, Jun Wang, Jiao Zhang, Yanfeng Han, Xi Li

**Affiliations:** 1 Shanghai Key Laboratory of Advanced High-temperature Materials and Precision Forming, Shanghai Jiao Tong University, Shanghai 200240, China; E-Mails: tclx8410104@sjtu.edu.cn (X.L.); zj119@sjtu.edu.cn (J.Z.); yfhan@sjtu.edu.cn (Y.H.); 2 State Key Laboratory of Metal Matrix Composites, Shanghai Jiao Tong University, Shanghai 200240, China; 3 School of Material Science and Engineering, Shanghai University, Shanghai 200240, China; E-Mail: lll@shu.edu.cn

**Keywords:** directional solidification, crystal structure, magnetic fields, segregation

## Abstract

A Ni-based superalloy CMSX-6 was directionally solidified at various drawing speeds (5–20 μm·s^−1^) and diameters (4 mm, 12 mm) under a 0.5 T weak transverse magnetic field. The results show that the application of a weak transverse magnetic field significantly modified the solidification microstructure. It was found that if the drawing speed was lower than 10 μm·s^−1^, the magnetic field caused extensive macro-segregation in the mushy zone, and a change in the mushy zone length. The magnetic field significantly decreases the size of γ’ and the content of γ-γ’ eutectic. The formation of macro-segregation under a weak magnetic field was attributed to the interdendritic solute transport driven by the thermoelectric magnetic convection (TEMC). The γ’ phase refinement could be attributed to a decrease in nucleation activation energy owing to the magnetic field during solid phase transformation. The change of element segregation is responsible for the content decrease of γ-γ’ eutectic.

## 1. Introduction

Nickel-based superalloys have been used for more than 30 years as materials for blades in aerospace turbine engines. Mechanical properties of nickel-based superalloys are closely related to their solidification microstructure, which mainly involves macro-segregation, γ’, γ-γ’ eutectic. The size, morphology and distribution of the γ’ precipitates significantly decide the mechanical properties of superalloys [1]. The γ-γ’ eutectic should be restrained because it depletes the formation element of the γ’ phase and reduces the start melt temperature [2].

The application of a uniform magnetic field in the area of solidification has attracted increasing attention [3,4,5,6]. It has been shown that the convection flows in a planar solid/liquid interfacial area can be significantly reduced by applying a magnetic field in the melts. For example, Boettinger *et al*. [7] found that a 0.1 T axial magnetic field applied in the directional solidification of Pb-Sn alloys reduces the macro-segregation resulting from solute convection. However, Lehmann *et al*. [8] indicated that a new convection named the thermoelectric magnetic convection is induced by the uniform magnetic field applied in the directional solidification. Li *et al*. [9] found the thermoelectric magnetic convection induced by the interaction between the thermoelectric current and the magnetic field caused the deflection of the liquid-solid interfaces and extensive segregations (*i.e*., freckles and channels) in the mushy zone. The effect of the magnetic field enhanced when the drawing speed decreased.

In recent years, the uniform magnetic field has been gradually applied in the directional solidification of Ni-based superalloys. Ren *et al*. [10] and Zhang *et al*. [11] found that a high magnetic field can significantly influence the primary dendrite arm spacing and segregation of DZ417G Ni-based superalloy. However, how the applied magnetic field influences the solidification microstructures of Ni-based superalloys, especially the macro-segregation and precipitation phases, is still far from being completely understood. Previous work [12] investigated the effect of a 0.5 T axial magnetic field on the solidification structure during directional solidification. It was found that the magnetic field significantly affected the primary dendrite arm spacing of the superalloy when the drawing speed was 20 μm·s^−1^. In this study, the influence of a weak transverse magnetic field on the directional solidification microstructure of Ni-based superalloy CMSX-6 was investigated in order to further understand the convections under the magnetic field and the effect of the produced convection on the solid/liquid interface.

## 2. Results

**Figure 1:**
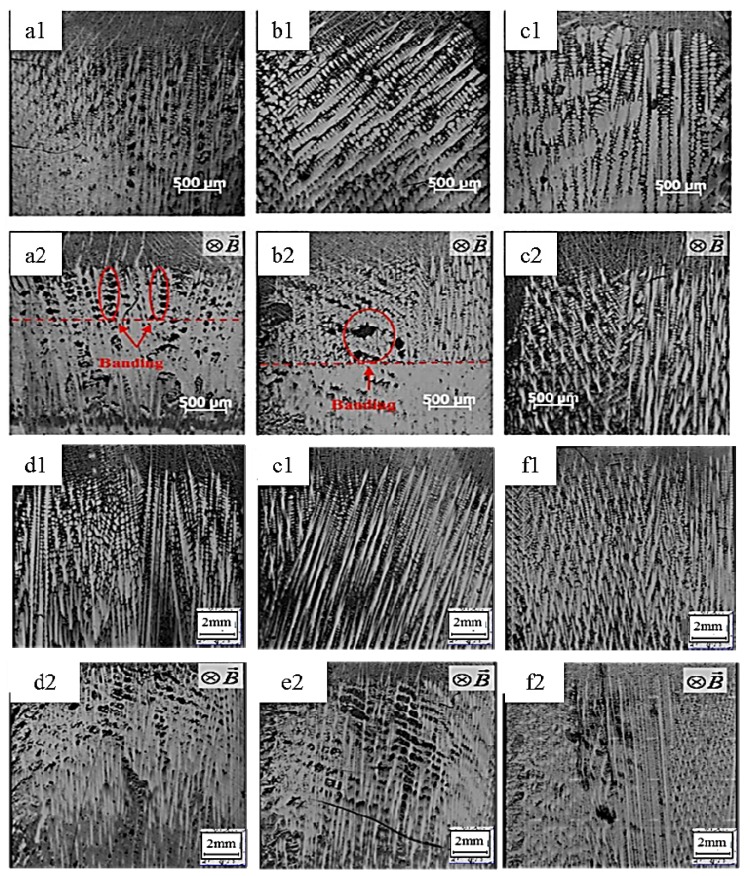
Longitudinal microstructure of directionally solidified superalloy at a temperature gradient of 80 K·cm^−1^ without and with a 0.5 T transverse magnetic field ($\vec{Β}$): (**a1**) 0 T, 5 μm·s^−1^, diameter of 4 mm; (**a2**) 0.5 T, 5 μm·s^−1^, diameter of 4 mm; (**b1**) 0 T, 10 μm·s^−1^, diameter of 4 mm; (**b2**) 0.5 T, 10 μm·s^−1^, diameter of 4 mm; (**c1**) 0 T, 20 μm·s^−1^, diameter of 4 mm; (**c2**) 0.5 T, 20 μm·s^−1^, diameter of 4 mm; (**d1**) 0 T, 5 μm·s^−1^, diameter of 12 mm; (**d2**) 0.5 T, 5 μm·s^−1^, diameter of 12 mm; (**e1**) 0 T, 10 μm·s^−1^, diameter of 12 mm; (**e2**) 0.5 T, 10 μm·s^−1^, diameter of 12 mm; (**f1**) 0 T, 20 μm·s^−1^, diameter of 12 mm; (**f2**) 0.5 T, 20 μm·s^−1^, diameter of 12 mm.

**Figure 2:**
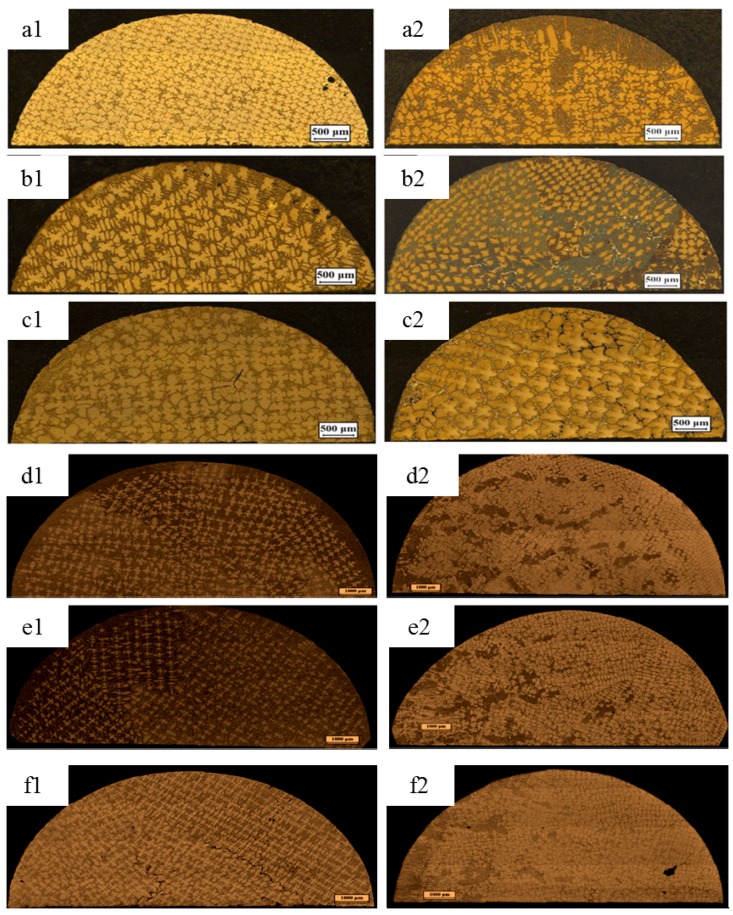
Transverse microstructure of directionally solidified superalloy at a temperature gradient of 80 K·cm^−1^ without and with a 0.5 T transverse magnetic field ($\vec{Β}$): (**a1**) 0 T, 5 μm·s^−1^, diameter of 4 mm; (**a2**) 0.5 T, 5 μm·s^−1^, diameter of 4 mm; (**b1**) 0 T, 10 μm·s^−1^, diameter of 4 mm; (**b2**) 0.5 T, 10 μm·s^−1^, diameter of 4 mm; (**c1**) 0 T, 20 μm·s^−1^, diameter of 4 mm; (**c2**) 0.5 T, 20 μm·s^−1^, diameter of 4 mm; (**d1**) 0 T, 5 μm·s^−1^, diameter of 12 mm; (**d2**) 0.5 T, 5 μm·s^−1^, diameter of 12 mm; (**e1**) 0 T, 10 μm·s^−1^, diameter of 12 mm; (**e2**) 0.5 T, 10 μm·s^−1^, diameter of 12 mm; (**f1**) 0 T, 20 μm·s^−1^, diameter of 12 mm; **(f2)** 0.5 T, 20 μm·s^−1^, diameter of 12 mm.

**Figure 3:**
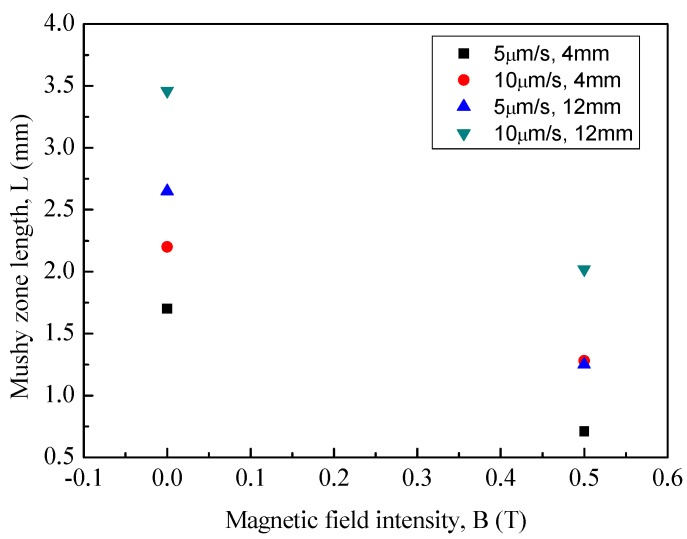
Mushy zone length as a function of magnetic field intensity.

Figure 1 shows the influence of a 0.5 T magnetic field on the longitudinal microstructures of superalloy CMSX-6 with diameter of 4 mm and 12 mm directionally solidified at a temperature gradient of 80 K·cm^−1^ and at various drawing speeds. It is observed that the typical dendritic crystals grow and regularly align without the magnetic field as shown in Figure 1a1–f1. When a 0.5 T magnetic field is applied in the solidification, the appearance of banding-like macro-segregation is induced. It was also found that the quantity of channel macro-segregation depends on the drawing speed. The channel macro-segregation is small at a drawing speed of 5 μm·s^−1^ and diameter of 4 mm as shown in Figure 1a2. When the drawing speed is increased to 10 μm·s^−1^, the macro-segregation reaches the maximum as shown in Figure 1b2. Then the macro-segregation disappears if the drawing speed is further increased to 20 μm·s^−1^, as shown in Figure 1c2. In Figure 1d2–e2, with the application of a 0.5 T magnetic field in the solidification, some channel macro-segregation appears in the center of the specimen with the diameter of 12 mm and its quantity is gradually increased with increasing drawing speed. The transverse microstructures of superalloy CMSX-6 with a diameter of 4 mm and 12 mm directionally solidified as mentioned above are shown in Figure 2. As seen in the Figure 2a1–f1, without the magnetic field, the dendrite morphology is typically columnar. However, when a 0.5 T magnetic field is applied, channel macro-segregations form on the samples in Figure 2a2–f2. We can clearly find the appearance of channel macro-segregation when a 0.5 T magnetic field is applied. Furthermore, the macro-segregation in the samples with a diameter of 12 mm is much more severe than that in the samples with a diameter of 4 mm. Additionally, it was noticed that the length of mushy zone is decreased with application of the magnetic field. The length of mushy zone under various growth speeds and diameters as a function of the magnetic field intensity is shown in Figure 3.

**Figure 4:**
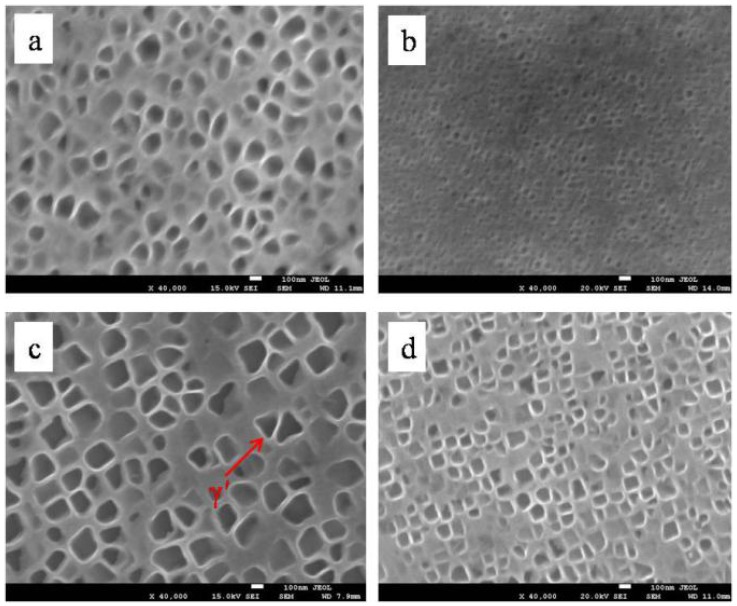
The morphology of γ’ of directionally solidified superalloy at a temperature gradient of 80 K·cm^−1^, drawing speed of 10 μm·s^−1^: (**a**) 0 T, diameter of 4 mm; (**b**) 0.5 T, diameter of 4 mm; (**c**) 0 T, diameter of 12 mm; (**d**) 0.5 T, diameter of 12 mm.

**Figure 5:**
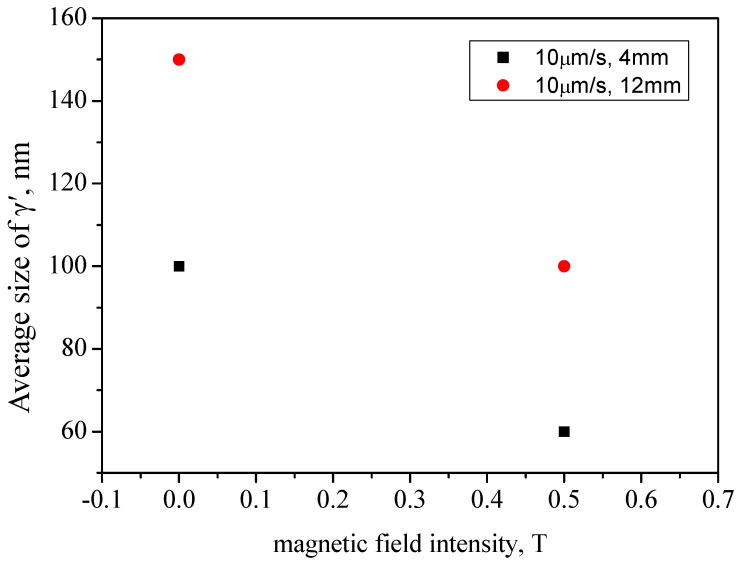
The γ’ size in the dendrite core of Ni-based specimens with various diameters at a temperature gradient of 80 K·cm^−1^.

Figure 4 shows the γ’ morphology in the dendrite core of specimens at the bottom of the liquid-solid mushy zone at a drawing speed of 10 μm·s^−1^ without and with 0.5 T magnetic field. It can be seen that the magnetic field dramatically reduces the size of the γ’ phase. Figure 5 shows the γ’ size of specimens with 10 μm·s^−1^ drawing speed and various magnetic field intensities. Compared to that without magnetic field, the γ’ size in the samples at diameters of 4 mm and 12 mm with 0.5 T magnetic field is decreased by 40% and 33%, respectively.

**Figure 6:**
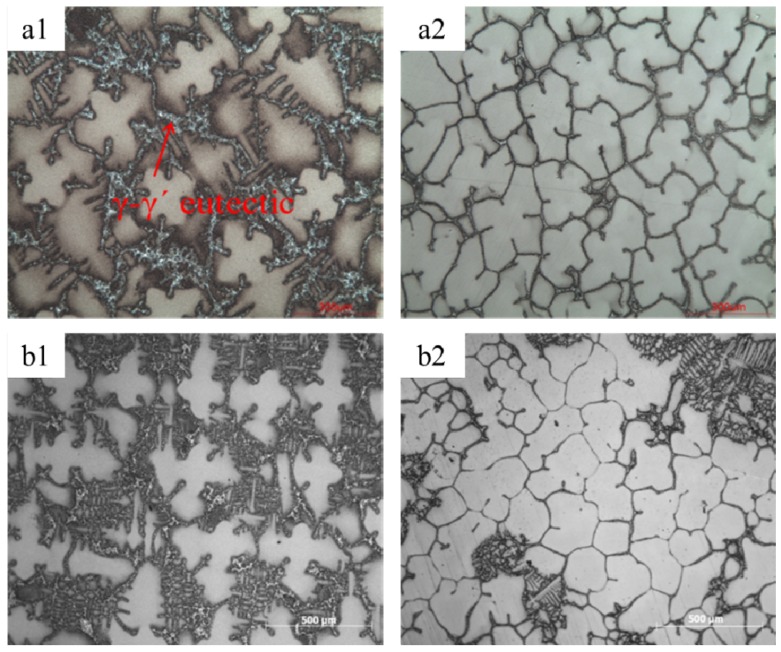
The morphology of γ-γ’ of directionally solidified superalloy at a temperature gradient of 80 K·cm^−1^, drawing speed of 10 μm·s^−1^: (**a1**) 0 T, diameter of 4 mm; (**a2**) 0.5 T, diameter of 4 mm; (**b1**) 0 T, diameter of 12 mm; (**b2**) 0.5 T, diameter of 12 mm.

**Figure 7:**
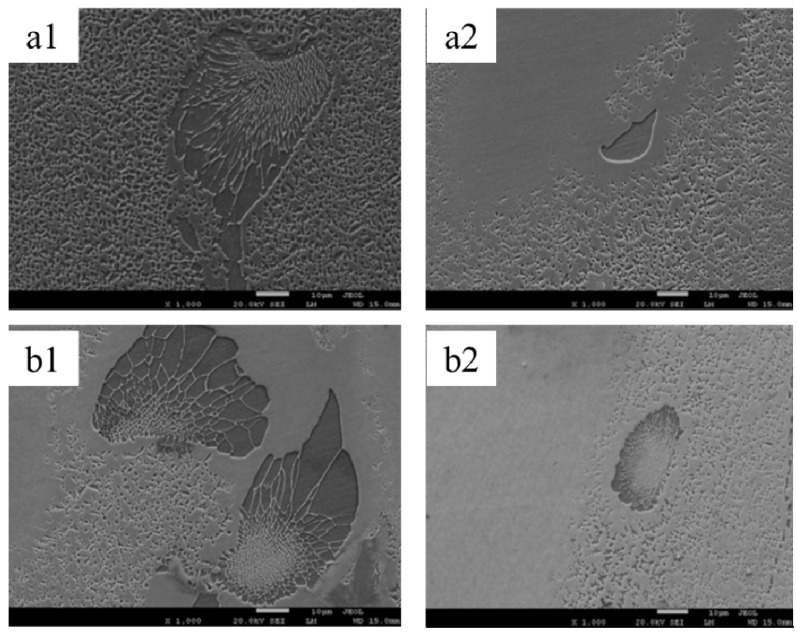
Scanning electron microscopy (SEM) image revealing the morphology of γ-γ’ eutectic of directionally solidified superalloy at a temperature gradient of 80 K·cm^−1^, drawing speed of 10 μm·s^−1^: (**a1**) 0 T, diameter of 4 mm; (**a2**) 0.5 T, diameter of 4 mm; (**b1**) 0 T, diameter of 12 mm; (**b2**) 0.5 T, diameter of 12 mm.

**Figure 8:**
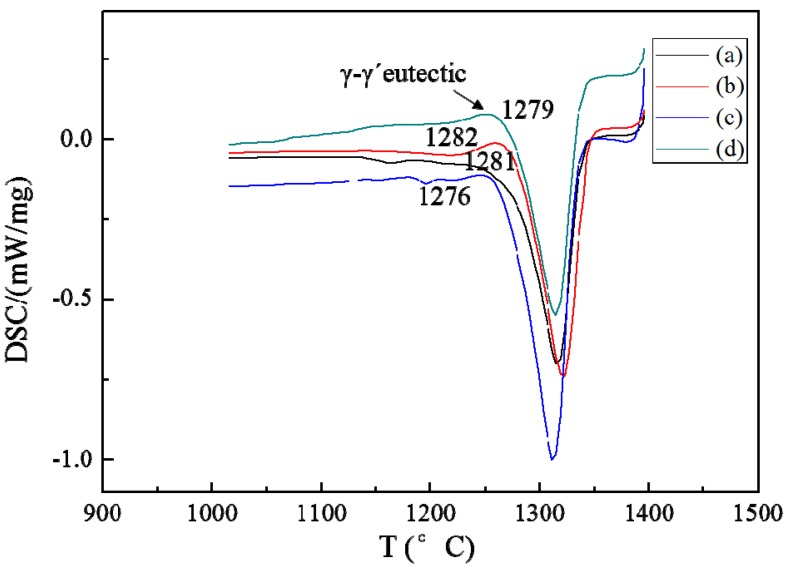
DSC heating curve (1000–1400 °C, 20 °C·min^−1^) of directionally solidified superalloy at a temperature gradient of 80 K·cm^−1^, drawing speed of 10 μm·s^−1^: (**a**) 0 T, diameter of 4 mm; (**b**) 0.5 T, diameter of 4 mm; (**c**) 0 T, diameter of 12 mm; (**d**) 0.5 T, diameter of 12 mm.

Another striking modification by the magnetic field is the decrease of eutectic volume. Either metallographic observation or DSC test confirms the decrement of the eutectic phase. Figure 6 shows the transverse microstructure of directionally solidified superalloy CMSX-6 with and without a 0.5 T magnetic field at a temperature gradient of 80 K·cm^−1^, drawing speed of 10 μm·s^−1^ and diameter of 4 mm and 12 mm. It can be observed that γ-γ’ eutectic is dispersed in the interdendritic regions without magnetic field. With the application of 0.5 T magnetic field, the quantity of γ-γ’ eutectic phase is significantly decreased. The γ-γ’ eutectic volume is reduced from 5.22% to 1.52% for the superalloy CMSX-6 of 4 mm in diameter as listed in Table 1. The effect of the magnetic field on the γ-γ’ eutectic phase was magnified when the diameter of the sample was expanded to 12 mm. The γ-γ’ eutectic volume in the sample was reduced from 5.72% to 1.95%. Figure 7 shows that at a temperature gradient of 80 K·cm^−1^, drawing speed of 10 μm·s^−1^ and diameter of 4 mm and 12 mm, the size of the γ-γ’ eutectic was decreased remarkably with a 0.5 T magnetic field. It was shown that a DSC test could confirm the change of eutectic [13]. As shown in Figure 8, both liquidus and solidus are encouraged slightly with the magnetic field; however, the solidification interval is not affected remarkably.

**Table 1:** Influence of magnetic field on eutectic formation of directionally solidified superalloy at a temperature gradient of 80 K·cm^−1^, drawing speed of 10 μm·s^−1^.

Samples	Area fraction (%)
0 T, diameter of 4 mm	5.22
0.5 T, diameter of 4 mm	1.52
0 T, diameter of 12 mm	5.72
0.5 T, diameter of 12 mm	1.95

## 3. Discussion

### 3.1. Effect of a Transverse Magnetic Field on the Liquid–Solid Interface Shape and Macrosegregation

**Figure 9:**
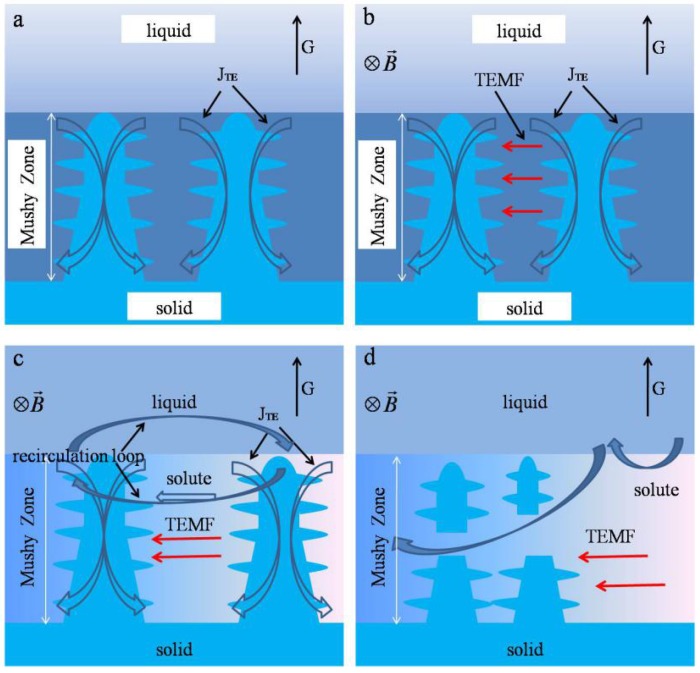
Schematic illustration of thermoelectric magnetic convection of transverse magnetic field. (**a**) Schematic views of TE effects during directional solidification under a magnetic field; (**b**) Schematic views of thermoelectric magnetic force; (**c**) recirculation loop induced by TEMC; (**d**) macro-segregation formed.

The above experimental results show that the application of a transverse magnetic field during the directional solidification of Ni-based superalloy can trigger the formation of macro-segregation. This could be attributed to the effect of convection on the solute distribution in the interdendritic area. In the process of directional solidification, the temperature at the tip of a dendrite is higher than that at the bottom, which generates a temperature gradient (G) along the longitudinal direction of dendrite. Accordingly, a thermoelectric (TE) current and a thermoelectric current J_TE_ on the dendrite are produced as shown in Figure 9a. When a transverse magnetic field is applied, a thermoelectric magnetic force (TEMF) is induced. Owing to the TEMF, a thermoelectric magnetic convection (TEMC) directionally appears in the interdendritic area with the application of a transverse magnetic field as shown in Figure 9b. The TEMC will further induce a recirculation loop in the bulk melt, ahead of, and in the mushy zone. The TEMC and the corresponding recirculation loop force the solute in the interdendritic area to move in a unique direction as shown in Figure 9c. Accompanying the moving solute, the density difference arises with lower density in the interdendritic fluid than that at top of the dendrite, which results in a convection. The lower dense solute in the interdendritic fluid is advected out of the interdendritic region, and the downward flow brings higher temperature liquid from the bulk into the mush zone. The higher temperature liquid from the bulk re-melts the dendrite and changes the temperature gradient of the area of mushy zone, so that macro-segregation forms and the length of mushy zone decreases as shown in Figure 9d.

By performing a mass balance on a small mushy volume element that contains both solid and liquid, a modified Scheil equation involving the possible in-flow/out-flow of liquid and the different densities of the two phases can be derived as follows [14]:

(1) $$\frac{\partial f_{L}}{\partial T}=-\left(\frac{1-\beta }{1-k}\frac{f_{L}}{C_{L}}\frac{\partial C_{L}}{\partial T}\right)\left(1+\frac{\vec{V}\cdot \nabla T}{\varepsilon }\right)$$

where $f_{L}$ is the fraction of liquid; $C_{L}$ is the solute concentration in the liquid; $\beta =\frac{\rho_{S}-\rho_{L}}{\rho_{S}}$ is the solidification shrinkage with $\rho_{L}$ and $\rho_{S}$ being the densities of the liquid and solid, respectively; k is the partition coefficient; is the velocity vector of the liquid; Δ*T* is the temperature gradient; and ε is the rate of temperature change.

The physical significance of Equation (1) can be understood as follows: when $\frac{\partial f_{L}}{\partial T}$> 0, no macro-segregation is produced; if $\frac{\partial f_{L}}{\partial T}$ < 0, macro-segregation will appear.

In $\left(\frac{1-\beta }{1-k}\frac{f_{L}}{C_{L}}\frac{\partial C_{L}}{\partial T}\right)$ if *k* < 1, $\frac{\partial f_{L}}{\partial T}$ < 0; when *k* > 1, $\frac{\partial C_{L}}{\partial T}$ > 0. So the value of this equation is always negative. Thus, if macro-segregation will form, $\frac{\partial C_{L}}{\partial T}$ < 0 and $(1+\frac{\bar{V}\cdot \nabla T}{\varepsilon })<0$. That is:

(2) $$\frac{\vec{V}\cdot \nabla T}{\varepsilon }<-1$$

As we know, the rate of temperature change, ε, in the directional solidification, is estimated by:

(3) $$\varepsilon =\nabla T\cdot \vec{R}$$

where Δ*T* is the temperature gradient; is the drawing speed. We set Equation (3) into Equation (2):

(4) $$\frac{\vec{V}}{\vec{R}}<-1$$

The Equation (4) shows that the formation of macro-segregation in the mush zone is decided by the ratio of the velocity vector of the liquid, $\vec{V}$, to the drawing speed, $\vec{R}$. Therefore, in the above experimental results, the speed of TEMC [12], *u*_1_, with a 0.5 T magnetic field is:

(5) u1≈(σSGBλρ)12

where σ, *S*, *B*, λ and ρ, respectively, denote the electrical conductivity, the absolute thermoelectric power of the conducting medium, the applied magnetic field, the typical length scale and the density. Then the velocity vector of the liquid $\vec{V}$ is:

(6) $${\vec{V}}_{1}=\vec{V}+{\vec{u}}_{1}$$

From Equations (4) and (6), it can be deduced that the application of the magnetic field during the directional solidification may enhance the velocity of the liquid and the possibility of the formation of macro-segregation.

When the intensity of the magnetic field is definite, the drawing speed is highlighted. According to the present experimental results, when the drawing speeds are 5 μm·s−1 and 10 μm·s−1, it can be estimated that $\frac{\vec{V}}{\vec{R}}<-1$, we can find macro-segregation. As the diameter of the sample increases from 4 mm to 12 mm, the speed of TEMC *u*_1_ increases too. Furthermore the effect of convection in the bulk melt ahead of and in the mushy zone is enhanced as the thermoelectric magnetic convection becomes stronger. Therefore the segregation in the samples with a diameter of 12 mm is much more severe than that in the samples with a diameter of 4 mm.

### 3.2. Effect of a Transverse Magnetic Field on Precipitation Phases

The nucleation activation energy of the γ′ phase precipitates is [15]:

(7) $$\Delta G^{*}=(16\pi \sigma_{\gamma -\gamma^{'}}^{3})/3\Delta G_{V}^{2}$$

where $\sigma_{\gamma -\gamma^{,}}$ is the interface energy between γ′ and γ phase, $\Delta G_{V}$ is the volume free energy difference. The critical radius is given by:

(8) $$r^{*}=2\sigma_{\gamma -\gamma^{'}}/\Delta G_{V}$$

The $\Delta G_{V}$ in the γ′ phase precipitation can be expressed as:

(9) $$\Delta G_{V}=\Delta S_{V}\Delta T$$

where $\Delta S_{V}$ is the entropy change per unit volume, ΔT is the undercooling value. When a magnetic field was applied, we found a decreased mushy zone length and assumed this was because the TEMC and the recirculation loop, which the TEMC induced, brought the higher temperature liquid from the bulk into the mush zone, and then changed the temperature gradient of the area of the mushy zone. It can be deduced that the application of the magnetic field during directional solidification may increase the temperature gradient (ΔT) [9]. Also, we can further reason that the application of the magnetic field could increase the undercooling values (ΔT). So, the $\Delta G_{V}$ is increased. From Equations (5) and (6), it can be deduced that when a magnetic field is applied, the nucleation activation energy and the critical radius of the γ’ phase are both decreased. Thus the quantity of γ’ phase is increased, and the size of γ’ is decreased.

**Figure 10:**
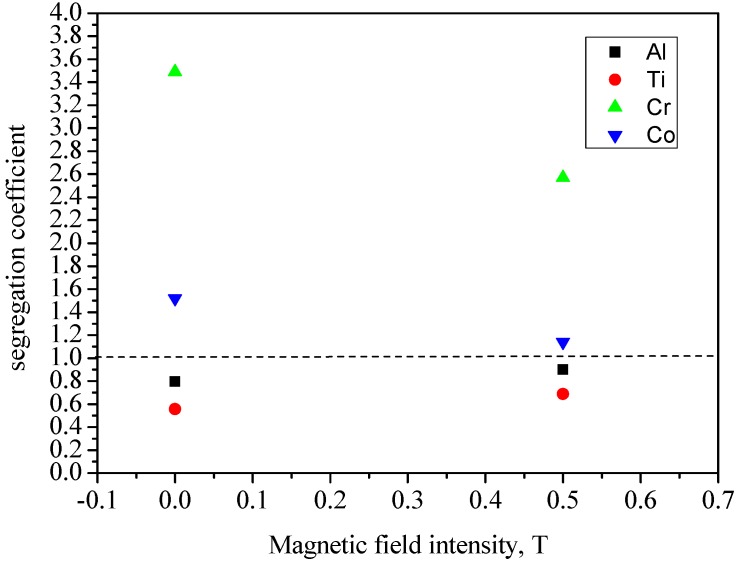
The dependence of segregation coefficient of Ni-based specimens on magnetic field at a temperature gradient of 80 K·cm^−1^, drawing speed of 10 μm·s^−1^, diameter of 12 mm.

Figure 10 presents the element segregation coefficient *k*’ of single crystal specimens at a temperature gradient of 80 K·cm^−1^, drawing speed of 10 μm·s^−1^, diameter of 12 mm as a function of intensities of magnetic field. Al and Ti show positive segregation behavior (*k*’ < 1), and Cr and Co show negative segregation behavior (*k*’ > 1), which agrees with that in Reference [15]. At the 0.5 T magnetic field, *k*’ of Al and Ti increase by 11% and 13%, respectively, and *k*’ of Cr and Co decrease by 27% and 25%, respectively. This indicates that the magnetic field dramatically reduces alloying element segregation. The element segregation affects the amount of γ-γ’ eutectic. The less segregation, the less amount of γ-γ’ eutectic. Since the magnetic field dramatically decreases the element segregation, a decrease in content of γ-γ’ eutectic will be obtained.

## 4. Experimental Section

The chemical composition of the used Ni-based superalloy CMSX-6 in this study is 0.0032 wt% C, 10.10 wt% Cr, 2.99 wt% Mo, 4.95 wt% Co, 1.98 wt% Ta, 4.84 wt% Al, 4.72 wt% Ti, 0.021 wt% Fe, 0.0018 wt% S, 0.056 wt% Si, 0.01 wt% Mn, 0.088 wt% Hf, 0.0006 wt% Mg and Ni as balance. The raw alloy was melted in an induction furnace under vacuum conditions and cast into an ingot with a diameter of 200 mm. The directional solidification samples with 4 mm and 12 mm in diameter and 80 mm in length, were electro-discharge machined from the cast ingots. Then they were enveloped in high purity corundum tubes with inner diameters of 4 mm and 12 mm and length of 100 mm.

The directional solidification was carried out in a Bridgman-Stockbarge type furnace. The temperature in the furnace was controlled with a precision of ±0.1 °C. A water-cooled cylinder containing liquid Ga-In-Sn metal (LMC) was used to cool down the specimen. The temperature gradient in the specimen was controlled by adjusting the temperature of the hot zone in the furnace, which was insulated from the LMC by a corundum disc. The static superconductor magnet used could produce a horizontal static magnetic field with a maximum adjustable intensity of 0.7 T.

During the experiment, the samples in the corundum crucibles were melted at 1500 °C and directionally solidified in the Bridgman apparatus by pulling the crucible assembly into the LMC cylinder at various velocities. After 60 mm steady-state growth of the specimen, the quenching experiment was carried out by quickly withdrawing the crucible into the LMC cylinder. The temperature gradient was 80 K·cm^−1^, and the drawing speed were 5, 10, 20 μm·s^−1^ in this study. The specimen for microstructure analysis was polished, etched in a solution of HCl and H_2_O_2_ at a proportion of 1:1. Then the microstructure was observed on an Imager A1m optical microscope (Zeiss, Oberkochen, Germany) and JSM7600F scanning electron microscope (SEM) (JEOL, Tokyo, Japan). The compositions of these minor phases were investigated via an energy dispersive X-ray spectroscope (EDS) (JEOL, Tokyo, Japan). The eutectic volume was counted using the DT2000 V2.0 image analysis system (DT East Image, Nanjing, China). The differential scanning calorimeter (DSC) of NETZSCH DSC404 (NETZSCH, Bavarian Asia, Germany) was used for thermal analysis. The DSC error of measurements of the equipment we used was ±0.2 °C.

## 5. Conclusions

The influence of a 0.5 T weak transverse magnetic field on the morphology of the liquid-solid interface was investigated during Bridgman growth of a Ni-base superalloy.

1) The magnetic field caused extensive macro-segregation in the mushy zone, and a change in the mushy zone length. The drawing speed and the diameter of specimen variation can affect the influence of the magnetic field.
2) The size of γ’ decreased by 40% and 33% with 0.5 T magnetic field at a diameter of 4 mm and 12 mm, respectively. The content of γ-γ’ eutectic was also diminished with a 0.5 T magnetic field.
3) The formation of macro-segregation under a weak magnetic field was attributed to TEMC-driven interdendritic solute transport. The refinement of the γ’ phase could be attributed to a decrease in nucleation energy. The content decrease of γ-γ’ eutectic results from the reduction of element segregation.
